# Application of Novel Biomaterials to Enhance Bone Regeneration in a Canine Non-Union Olecranon Fracture

**DOI:** 10.3390/ani15202968

**Published:** 2025-10-14

**Authors:** Taeseok Noh, YoungJin Jeon, Se-Heang Oh, Sunglim Lee, Yoonho Roh

**Affiliations:** 1 Department of Veterinary Surgery, College of Veterinary Medicine, Gyeongsang National University, Jinju 52828, Republic of Korea; nots1300@gnu.ac.kr; 2 Department of Veterinary Surgery, College of Veterinary Medicine, Chungnam National University, Daejeon 34134, Republic of Korea; orangee0115@cnu.ac.kr; 3 Department of Biomedical Sciences and Biosystems, Dankook University, 119 Dandae-ro, Dongnam Gu, Cheonan 31116, Republic of Korea; seheangoh@dankook.ac.kr; 4 Department of Veterinary Obstetrics and Theriogenology, College of Veterinary Medicine, Gyeongsang National University, Jinju 52828, Republic of Korea; sllee@gnu.ac.kr

**Keywords:** non-union fracture, recombinant human bone morphogenetic protein-2, leaf-stacked structure, mesenchymal stem cell

## Abstract

**Simple Summary:**

A “non-union” fracture is a broken bone that does not heal over a long period of time, regardless of whether surgery was performed or not. This type of fracture is very difficult to treat because the bone is both unstable and loses its ability to repair itself. In this report, we describe the treatment of a small dog that had lived with an untreated elbow fracture for two years. The dog could not use the forelimb and had severe stiffness and weakness. Surgeons used three-dimensional computer modeling to plan the operation and chose the most suitable plate to hold the bone together. In addition to fixing the bone with surgery, a new material made of very small, thin, leaf-stacked structures of polycaprolactone was placed into the fracture. These particles were combined with recombinant human bone morphogenetic protein-2 that stimulate bone growth and with stem cells, which are special cells that can develop into new bone. Over the following months, the dog regained the ability to walk normally and the bone healed, as shown on follow-up X-rays. This case shows that combining precise surgery with innovative biological materials can restore limb function in difficult cases, offering promise for animal health care.

**Abstract:**

A six-year-old, neutered male Pomeranian weighing 4.25 kg was presented with a two-year history of non-weight-bearing lameness of the left thoracic limb following an untreated traumatic olecranon fracture. Orthopedic examination revealed markedly reduced elbow joint range of motion and muscle atrophy. Radiographs demonstrated a distinct fracture line with proximolateral displacement of the olecranon fragment. Preoperative computed tomography (CT) and three-dimensional (3D) reconstruction were used to establish the surgical plan and to pre-contour a locking plate. Surgical treatment was performed in sequential steps, including removal of scar tissue, reopening of the bone marrow channel, and internal fixation. Considering the compromised biological environment of a chronic non-union, a bioactive graft composed of porous leaf-stacked structure (LSS) polycaprolactone particles incorporating recombinant human bone morphogenetic protein-2 (rhBMP-2) and mesenchymal stem cells (MSCs) was applied in combination with plate-screw fixation. The patient showed progressive improvement after surgery, achieving full weight-bearing and restoration of elbow joint motion comparable to the contralateral side. Follow-up radiographs and CT confirmed fracture union, and the radiolucency of the LSS scaffold enabled precise monitoring of bone healing. This case highlights the potential utility of combining patient-specific surgical planning with sustained delivery of rhBMP-2 and MSCs using LSS particles for the management of chronic non-union fractures in small animals.

## 1. Introduction

Olecranon fractures are commonly encountered in canine patients, typically resulting from traumatic incidents such as vehicular accidents or falls [1,2]. These fractures account for approximately 10% of all elbow fractures in small animals [1,2]. When displacement of the fracture margins is present, surgical intervention is essential to achieve anatomical reduction and stabilize the fracture through internal fixation [1,2,3]. Due to the functional significance of the triceps muscle, which inserts on the olecranon and generates considerable biomechanical forces during movement, inadequate stabilization of this region may result in persistent instability and eventual development of non-union [2,4,5,6]. Non-union fractures are reported to occur in approximately 3.4% to 8.1% of all fractures, although this rate may vary depending on individual patient factors [6,7]. In Veterinary Medicine, the etiology of non-union fractures typically involves failure of the mechanical environment, failure of the biological environment, or the presence of infection [5,6,7]. Mechanical failure may arise from either excessive motion at the fracture site or overly rigid fixation, both of which can compromise healing [6,7,8,9]. The failure of the biological environment can occur due to insufficient vascular development at the fracture site, leading to an inadequate supply of factors necessary for osteogenesis [6,7,8,9].

Treatment of non-union fractures generally requires a dual approach aimed at improving both the mechanical and biological environments [6]. To enhance the biological environment for fracture healing, commonly employed methods include debridement of fibrous tissue surrounding the fracture site and opening of the medullary canal to improve vascular supply [5,6,9]. However, even when adequate vascular access is achieved, the release of endogenous osteogenic proteins may be insufficient for effective healing [5,6,9]. To address this, biological agents such as mesenchymal stem cells (MSCs), recombinant human bone morphogenetic protein-2 (rhBMP-2), and various synthetic biomaterials have been investigated as adjunctive regenerative therapies [5,6,9]. RhBMP-2, in particular, has been increasingly utilized in Veterinary Medicine for the treatment of bone defects and non-union fractures [10,11,12]. More recently, research has focused on developing effective carriers capable of providing sustained and controlled release of osteoinductive agents, such as rhBMP-2, at the fracture site to enhance bone regeneration [13,14].

This case report presents a novel approach for managing a chronic non-union olecranon fracture using a synthetic bone graft material with a unique leaf-stacked structure (LSS), combined with rhBMP-2 and MSCs. This system could be a viable option to promote successful bone healing through sustained delivery of biological factors [14,15,16,17,18].

## 2. Case Presetation

### 2.1. Patient Information, Clinical Signs, and Physical Examination

A 6-year-old, 4.25 kg, neutered male Pomeranian was presented with a history of persistent lameness following a fall two years ago. Because of the owner’s situation, the dog did not receive surgical treatment at that time. Two years after the trauma, the patient was presented to Gyeongsang National University Animal Medical Center for the treatment. The patient had been non-weight-bearing on the left forelimb for two years and exhibited a lameness score of 5/5 during gait evaluation [19]. Upon physical examination, the dog was alert and a body condition score (BCS) of 5/9. Vital signs, including blood pressure, heart rate, and respiratory rate, were within normal limits. Blood tests revealed no abnormal findings. Orthopedic examination revealed a normal range of motion (ROM) in the right elbow joint (45–165°), while restricted ROM in the left elbow joint (25–95°), consistent with limited extension and joint dysfunction. A firm bony prominence was noted on the lateral aspect of the elbow joint with the suspected olecranon palpated as shorter compared to the right side. Muscle mass was assessed using a Gulick tape at the midshaft of the humerus and at the proximal one-third of the radius. In the right forelimb, the circumference measured 118 mm at the humeral midshaft and 82 mm at the proximal radius, whereas in the left forelimb, the corresponding measurements were 94 mm and 71 mm, respectively. The differences between two limbs were calculated as percentages 79.16% and 88.89%, respectively. These results indicated that the left forelimb exhibited muscle atrophy compared with the right. Neurological examination was unremarkable.

### 2.2. Diagnostic Imagings

**Figure 1:**
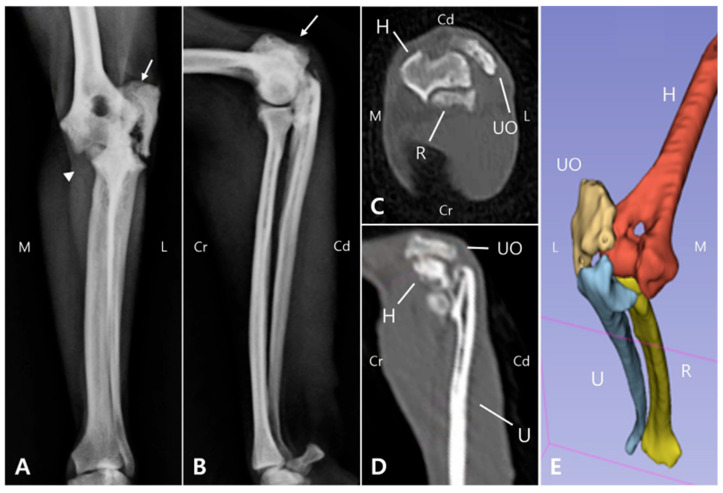
Preoperative radiographic, CT, and 3D reconstruction regarding the left elbow joint. On the anteroposterior radiograph (**A**), an olecranon fracture (arrow) and apparent dislocation of medial coronoid process (arrowhead) are evident. On the medio-lateral radiograph (**B**), the olecranon is displaced proximally (arrow), likely due to traction exerted by the triceps muscle. These images demonstrate a left olecranon fracture with associated ulnohumeral luxation. In the coronal and sagittal CT planes (**C** and **D**, respectively), periarticular fibrosis together with soft tissue proliferation at the fracture site impeded precise delineation of the fracture anatomy and localization. The 3D model (**E**) reconstructed from the CT data provides a clear structural depiction of the olecranon fracture and ulnar luxation. (M: Medial, L: Lateral, Cr: Cranial, Cd: Caudal, UO: Ulna olecranon, H: Humerus, U: Ulna, R: Radius).

Radiographic and computed tomography (CT) imaging were obtained, revealing a fracture involving the olecranon of the left ulna, with ill-defined fracture margins and ulna luxation, while radiocapitellar alignment remained preserved. Additionally, sclerosis, osteolysis, deformation, and craniolateral displacement of the olecranon were observed. Sclerosis was also observed at the proximal diaphysis of the left ulna, with a well-defined, linear 3.0 mm bone fragment located caudally (Figure 1A,B). Soft tissue width was assessed on lateral radiographs by measuring soft tissue thickness at the midshaft of the humerus (brachium) and at the proximal one-third of the radius (antebrachium). In the right forelimb, the soft tissue width measured 48.69 mm at the brachium and 28.53 mm at the antebrachium, whereas in the left forelimb, the corresponding measurements were 32.25 mm and 21.04 mm, respectively. The values of the affected limb corresponded to 66.2% (brachium) and 73.7% (antebrachium) of those of the contralateral side. These results were consistent with the circumferential measurements and indicated muscle atrophy in the affected limb compared with the contralateral side. These findings indicated moderate muscle atrophy in the left forelimb compared to the contralateral side. Due to the chronic nature of injury, the fracture and luxation led to fibrosis of the surrounding joint tissues and chronic arthritis (Figure 1C,D), making it challenging to clearly identify the fracture anatomy and localization, which was important for surgical planning. Therefore, 3D modeling and printing were utilized to enable more precise preoperative planning.

### 2.3. Presurgical Plannings

To develop a surgical plan for olecranon fracture repair and joint reconstruction, 3D models were reconstructed from CT Digital Imaging and Communications in Medicine (DICOM) files using 3D Slicer 5.2.2 (Brigham and Women’s Hospital, Boston, MA, USA) (Figure 1E). The fractured olecranon and luxated elbow joint were then modeled using 3ds Max (Autodesk, San Francisco, CA, USA). Both the fractured and simulated repaired bone models were 3D printed using UV Tough resin and a 3D printer (Anycubic Photon M3 Max, Shenzhen Anycubic Technology Co., Ltd., Shenzhen, China) [20,21].

**Figure 2:**
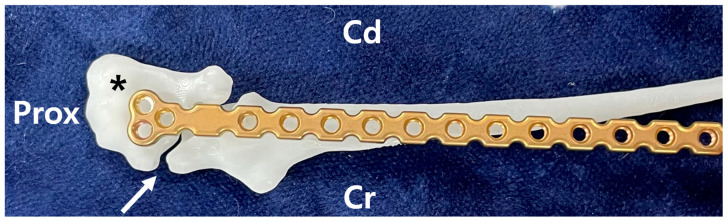
A T-shaped 1.2 mm locking plate of appropriate size was selected, allowing three screw holes in the proximal segment and three screw holes in the distal segment based on the 3D printed model. In addition, the fracture anatomy was confirmed using the 3D-printed model, including the poorly defined anconeal process (arrow) and the olecranon fragment (asterisk). (Cr: cranial, Cd: caudal, Prox: proximal).

Using the 3D-printed bones, a suitable plate for the fracture site was selected in advance of surgery. In this case, the fracture line was located at the most proximal end of the ulna, and the surgical plan involved placing the plate to the caudomedial aspect of the bone. The T-shaped 1.2 mm locking plate (Arix, Jeil medical corp., Seoul, Republic of Korea) was chosen, as it allowed for the placement of three screws in the olecranon and four screws into the ulna distal to the olecranon (Figure 2). The selected plate was contoured in advance based on the caudo-medial side of the repaired ulna 3D model to ensure optimal fit and alignment. Both the pre-contoured plate and the 3D models were plasma sterilized before surgery to maintain aseptic conditions. This approach facilitated the development of a precise surgical plan and ensured procedural efficiency.

### 2.4. Preparing Leaf-Stacked Structure (LSS)

**Figure 3:**
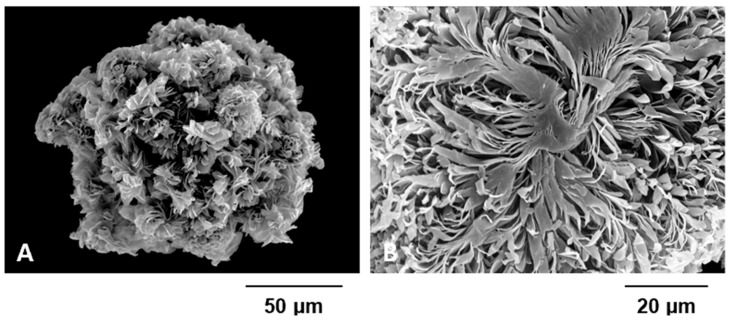
These figures show images of LSS particles observed using a scanning electron microscope (SEM; S-4300, Hitachi). The surface image (**A**) and cross-sectional image (**B**) demonstrate the porous nature of the LSS particles.

Polycaprolactone (PCL) with a molecular weight of 80,000 Da (Evonik, Essen, Germany) and tetraglycol (Sigma-Aldrich, St. Louis, MO, USA) were used to fabricate LSS particles [14,15,16,17]. LSS particles were fabricated using a previously reported heat-cooling technique [14,15,16,18]. PCL powder was dissolved in tetraglycol at a concentration of 15% and heating to 90 °C [14,15,18]. The resulting solution was then cooled at 4 °C for 1 h to induce polymeric precipitation [14,15,16]. The precipitated polymer (LSS particles) was thoroughly washed with water, sieved to obtain particles within a size range of 100–300 μm, and subsequently freeze-dried [14,15,18]. The morphology of the LSS particles was examined using a scanning electron microscope (SEM; S-4300, Hitachi, Tokyo, Japan) at the Center for Bio-Medical Engineering Core Facility (Dankook University, Cheonan, Republic of Korea) (Figure 3A,B) [15,16,17,18]. The LSS particles underwent ethylene oxide sterilization [14,15,16]. To load rhBMP-2 onto the LSS particles, 50 mg of sterilized particles were immersed in 1 mL of Phosphate-Buffered Saline (PBS, pH ~7.4) containing 1 μg/mL of rhBMP-2 and 1% bovine serum albumin (BSA) [14,15,16,17]. The mixture was incubated at 4 °C for 3 h under gentle positive pressure to enhance protein infiltration into the porous structure [14,16,17]. Following incubation, excess solution was removed, and the particles were freeze-dried [15,16,17].

### 2.5. Preparing Mesenchimal Stem Cell (MSC)

Canine adipose-derived mesenchymal stem cells (cAMSCs) were obtained from an established stem cell bank, and the following description outlines the general procedure for establishing this bank. The bank was generated from abdominal fat of clinically healthy beagle dogs (*n* = 3), following the approval of the Animal Center for Biomedical Experimentation at Gyeongsang National University (Approval No. GNU-210329-M0033) [22,23]. The adipose tissues were finely minced and enzymatically digested using 0.1% collagenase type I in PBS at 37 °C for 1 h [22,23]. The resulting cell suspension was filtered and centrifuged, and the cell pellet was resuspended in Advanced Dulbecco’s Modified Eagle’s Medium (ADMEM) supplemented with 10% heat-inactivated fetal bovine serum (FBS), 200 nM L-glutamine, 100 IU/mL penicillin, and 100 μg/mL streptomycin [22,23]. Cultures were maintained at 37 °C in a humidified atmosphere containing 5% CO_2_. Non-adherent cells were removed during medium changes performed twice weekly [22,23]. Upon reaching 80–90% confluency, adherent cells were detached using 0.25% trypsin-EDTA and subcultured for further experiments [22,23]. Cells from passage 4 were used for seeding onto the (LSS) in this particular case.

### 2.6. Cell Seeding onto LSS

The LSS particles (10 mg) were placed in 24-well plates, and a cAMSC suspension [passage 4; 1 × 10^6^ cells in 1 mL ADMEM containing 10% FBS, 100 IU/mL penicillin, and 100 μg/mL streptomycin] was seeded onto the particles. The constructs were incubated for 12 h with gentle shaking at 50 rpm to ensure uniform cell adhesion in a humidified incubator (21% O_2_, 5% CO_2_, 37 °C). The resulting cell/LSS complexes were then collected, and a total of 4.5 × 10^6^ cells within the complexes were injected into the defect site during surgery.

### 2.7. Surgery

The patient was premedicated with Midazolam (0.2 mg/kg, IV; Bukwang Midazolam Inj., Bukwang Pharm, Seoul, Republic of Korea) as a sedative, Tramadol (5 mg/kg, IV; Tramadol HCl Inj., Shinpoong Pharm, Seoul, Republic of Korea) as an analgesic, Cefazolin (25 mg/kg, IV; Cefozol Inj., Hankook Korus Pharm, Chuncheon, Republic of Korea) as a prophylactic antibiotic, and Famotidine (1 mg/kg, IV; Gaster^®^ Inj., Donga ST, Seoul, Republic of Korea) as a gastrointestinal protectant. Anesthesia was induced with Alfaxalone (2 mg/kg, IV; Alfaxan^®^ Multidose, Zoetis, Parsippany-Troy Hill, NJ, USA), and maintained with Isoflurane (Ifran^®^ Liq., Hana Pharm, Seoul, Republic of Korea). From pre-anesthesia to intubation, the patient received 100% oxygen. During surgery, Tramadol (1 mg/kg/h), Lidocaine (2 mg/kg/h; Daihan Lidocaine HCl Hydrate Inj. 1%, Dai Han Pharm, Seoul, Republic of Korea), and Ketamine (0.6 mg/kg/h; Ketamine HCl Inj., Huons, Seongnam, Republic of Korea) were administered Via constant rate infusion (CRI) for analgesia. Ringer’s lactate solution was used as intravenous fluid therapy for a total of eight days during hospitalization.

**Figure 4:**
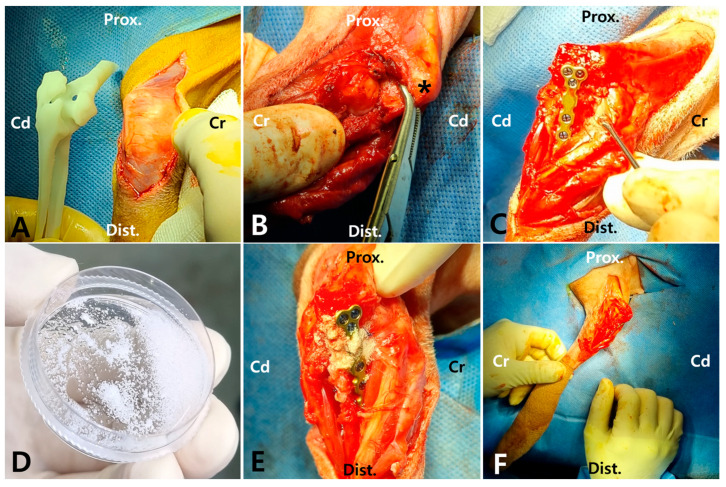
Surgical procedures including approach, fracture reduction, fixation, and application of the bioactive graft. The patient was positioned in right lateral recumbency, and a caudolateral approach was performed. (**A**) The skin was retracted medially and the fracture anatomy was confirmed with the assistance of a 3D-printed bone model. (**B**) After removal of periarticular fibrosis and opening of the medullary canal, the fracture was reduced by handling the olecranon (asterisk) with serrated bone-holding forceps toward the fracture site. (**C**) The fracture site was stabilized by placing a 1.2 mm T-plate and screws on the medial aspect of the ulna. (**D**) MSCs were combined with LSS particles preloaded with rhBMP-2. (**E**) The MSCs/LSS complexes were applied directly over the fracture site. (**F**) After stabilization, the gross lateral appearance of the skin incision made Via the caudolateral approach is depicted. (Cr = cranial; Cd = caudal; M = medial; Prox. = proximal; Dist. = distal).

The patient was positioned in right lateral recumbency, and the surgical incision was made Via a caudo-lateral approach, slightly lateral to the midline on the posterior aspect of the olecranon. (Figure 4A) To facilitate anatomical confirmation intraoperatively, a 3D model generated preoperatively from CT data was utilized for comparison and reference. (Figure 4B) The extensor carpi ulnaris and flexor carpi ulnaris muscles were elevated from the ulnar diaphysis and olecranon. Fibrous and proliferative tissue occupying the articular surface, which were corresponding to CT images, were meticulously removed using a periosteal elevator and monopolar electrocautery device. As the medullary cavity was covered by sclerotic tissue, multiple drill holes were made along the longitudinal axis of the fracture interfaces on both the proximal and distal segments using a 0.7 mm Kirschner wire to expose the medullary cavity. In addition, sclerotic tissue around the cortical bone at the fracture line was carefully debrided, while preserving the surrounding normal cortical bone. Before plate application, the olecranon was grasped with a serrated forceps to reduce the fracture (Figure 4B). Stabilization of the fracture was achieved using a pre-contoured 1.2 mm locking T-plate (head width 7 mm, shaft width 4 mm, thickness 1.7 mm; Jeil Medical Corp, Seoul, Republic of Korea) and 1.2 mm locking screws (Jeil Medical Corp, Seoul, Republic of Korea), with three screws placed in the proximal fragment and four in the distal fragment (Figure 4C). Following plate fixation, the elbow joint exhibited a significantly improved range of motion, especially in extension. This improvement was observed intraoperatively after removal of periarticular fibrosis and surgical reduction of the olecranon fracture with associated ulnohumeral luxation. The Cell/LSS complexes (Figure 4D) containing 4.5 × 10^6^ MSCs and rhBMP-2-incorporated LSS were delivered into the fracture gap using a periosteal elevator (Figure 4E). Routine closure was performed.

### 2.8. Postoperative Management and Follow-Ups

The patient recovered smoothly from anesthesia. During hospitalization, famotidine (0.5 mg/kg IV, BID) was administered. Analgesia was maintained for two days using a CRI of tramadol (1 mg/kg/h), lidocaine (2 mg/kg/h), and ketamine (0.6 mg/kg/h). The soft bandage was applied to protect the surgical wound and assist with activity restriction. The patient was discharged uneventlfully after six days with instructions to restrict activity for six weeks.

**Figure 5:**
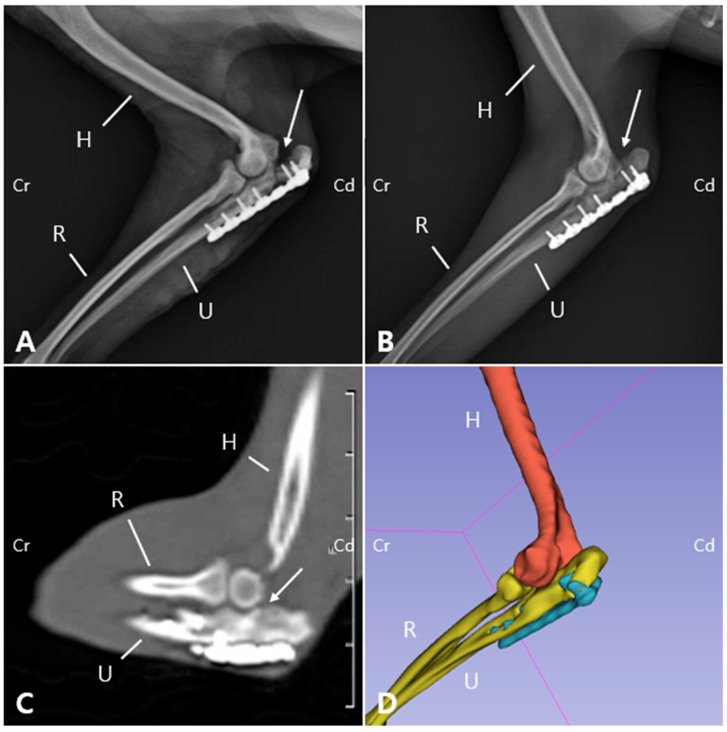
Sequential radiographic and CT evaluation of fracture healing following plate fixation. (**A**) Medial view radiographs obtained on the day of surgery show appropriate plate placement with a clearly visible fracture line (arrow). (**B**) At 140 days postoperatively, the fracture gap appears reduced (arrow). (**C**) On the CT scan acquired at POD 140, the previously distinct fracture line is no longer discernible. (**D**) The 3D reconstruction generated from the CT dataset revealed the absence of the anconeal process. (H: Humerus; U: Ulna; R: Radius; Cr: Cranial; Cd: Caudal).

Two weeks postoperatively, the sutures were removed. Follow-up evaluations were subsequently performed including physical and orthopedic examination, gross gait analysis and radiography. The bandage was maintained for 30 days postoperatively as a means of coaptation to limit excessive joint motion, given the articular nature of the fracture and the associated ulnohumeral luxation. Although a transarticular external fixator could have been considered, this option was not pursued due to management challenges and financial limitations. By postoperative day (POD) 50, the patient was able to bear weight on the left forelimb during walking, with a lameness score of 3/5, although mild weight shifting was still observed [19]. Radiographs revealed a visible fracture line but reduced sharpness of the margin of the ulnar trochlear notch (Figure 5A). On postoperative radiographs, the anconeal process was not clearly identifiable, which was consistent with the gross appearance of 3D printed models (Figure 2 and Figure 5). On POD 140, the owner reported that the patient was fatigued easily during walks but was able to walk normally and showed satisfactory recovery. Gait evaluation performed at the hospital exhibited a lameness score of 0/5, with no observable lameness during walking or trotting [19]. Additionally, no pain response was elicited during palpating or passive range of motion the joint. The ROM of the left elbow joint improved to 40–140°, comparable to that of the right elbow joint (45–165°), and revealing significant improvement compared to the initial presentation (25–95°). These findings suggest that functional preservation of the joint was successfully achieved. Radiographs and CT images acquired at POD 140 confirmed progressive reduction of the fracture line gap (Figure 5B,C). Three-dimensional modeling reconstructed from CT data further demonstrated the stable positioning of the implants and restoration of anatomical alignment (Figure 5D). Follow-up information obtained from the owner confirmed that, as of 483 days postoperatively, the patient was doing well without any evidence of forelimb lameness.

## 3. Discussion

This case report describes the first clinical application of a novel delivery system using LSS particles combined with 3D modeling based surgical planning. This approach was applied to concurrently address mechanical instability and a compromised biological environment in the treatment of a chronic non-union olecranon fracture in a 6-year-old Pomeranian that had remained untreated for over two years.

The incidence of non-union fractures in dogs is approximately 4.6%, with the radius and ulna being the most commonly affected sites, accounting for 40.6% of cases [6,7]. Revision surgeries for non-union fractures have demonstrated a high success rate of 94.7% [6,24]. Unlike previously reported cases, the patient in this case exhibited a chronic non-union fracture that had remained untreated for two years. This prolonged duration likely resulted in a compromised biological environment, unfavorable for bone healing. Two surgical options were considered for this case: Fracture reduction and internal fixation, and arthrodesis [4,6,9]. However, rather than immediately opting for joint fusion and thus sacrificing elbow function, fracture reduction was first attempted in order to preserve joint function as a more appropriate treatment approach. Fortunately, there were no signs of infection at the fracture site, which is a significant positive factor, considering that approximately 40% of non-union fractures are associated with infection [7]. Surgical intervention was undertaken based on the rationale that sustained local delivery of rhBMP-2 and MSCs using LSS particles [14,16,25], in conjunction with adequate internal fixation using a plate, would provide both the biological and mechanical conditions necessary for successful fracture healing.

The olecranon region of the ulna is subject to significant tensile forces due to the attachment of the triceps tendon [6,26]. In this case, the patient exhibited high activity levels and possessed extremely small bone fragments, necessitating a more robust fixation method to enhance the mechanical stability. A commonly used method for stabilizing such fractures involves inserting an intramedullary (IM) pin from the proximal to distal segment of the ulna combined with tension band wiring [4]. In small breed dogs, IM pins offer the advantages of reduced soft tissue trauma and ease of application [27,28,29]. Nonetheless, in the case of olecranon fractures, plate fixation provides superior stability under load-bearing conditions [27,28,29]. The limitation was not simply related to the patient’s size but to the pathological condition of the bone. The medullary cavity was occluded by sclerotic tissue, making intramedullary pinning—an essential component of tension band wiring—unfeasible, even after reopening with multiple drill holes created using a Kirschner wire. In addition, the compromised bone quality and the irregular fracture surface, which required partial debridement of sclerotic tissue and did not permit complete cortical contact, further reduced the suitability of this technique. Therefore, internal fixation with a small locking plate was considered a more reliable and stable option to achieve fracture stabilization [30,31].

To restore the severely compromised biological environment resulting from the two-year chronic non-union, the medullary cavity at the fracture site was re-opened using a K-wire. However, the extremely narrow canal and surrounding sclerosis limited the capacity to establish a favorable biological environment for healing, even after debridement. Because the intrinsic biological potential was limited, various strategies, including stem cell therapy, application of growth factors, biomaterials and tissue engineering, gene therapy, and modulation of signaling pathways were explored in previous studies [32,33,34,35,36]. Although various treatment strategies have been proposed, in this case LSS particles incorporating rhBMP-2 and mesenchymal stem cells were applied to provide sustained osteoinductive stimulation as well as an additional cellular source to enhance bone regeneration. The therapeutic benefits of rhBMP-2 and MSCs, both individually and in combination, are well documented in the veterinary and medical literature [25,37].

Conventional carriers for rhBMP-2, such as absorbable collagen sponges or hydroxyapatite-based substitutes, present limitations. Collagen matrices are associated with rapid burst release, whereas hydroxyapatite can obscure radiographic evaluation due to radio-opacity [14,15,16,33]. In contrast, LSS particles are composed of densely stacked, flat and elongated leaf-like structures that provide a large surface area for cell adhesion, proliferation, and differentiation, while also ensuring sustained release of bioactive molecules such as rhBMP-2 for up to 26 days [14,15,16,17,18]. This prolonged release is clinically relevant because it extends biological activity beyond the initial inflammatory phase, supporting bone formation throughout the repair and early remodeling phases of fracture healing. Additionally, the radiolucency of the LSS particles allowed clear visualization of the fracture line and bridging callus during postoperative follow-up, avoiding the interference commonly seen with opaque carriers such as hydroxyapatite.

In small-breed dogs with non-union fractures, previous studies have reported prolonged healing times, often exceeding a median of 13 weeks and in some cases extending to as long as 53 weeks [38,39,40]. In contrast, fracture union in this patient was radiographically and clinically confirmed by POD 140. Although direct comparisons across studies are limited, this relatively shorter healing period suggests that the combined use of rhBMP-2, MSCs, and LSS particles may have contributed to more efficient bone regeneration in an otherwise unfavorable biological environment. This case therefore highlights not only the feasibility but also the potential clinical utility of LSS-based delivery systems in managing challenging non-union fractures in small animals.

In cases of chronic elbow joint luxation in dogs, achieving complete anatomical reduction Via closed methods is often challenging [41,42]. Nevertheless, periarticular fibrosis and skeletal remodeling may still provide a functionally stable joint [38,39]. In the present case, intraoperative removal of periarticular fibrosis and fracture reduction resulted in a marked improvement in elbow range of motion, even though the anconeal process remained poorly defined. These findings indicated that stabilization of the fracture and restoration of anatomical alignment were sufficient to regain functional joint mobility, without the need for additional procedures such as arthrodesis or joint reconstruction.

Immediate postoperative radiographs, CT, and CT-based 3D reconstructions confirmed that the plate was securely placed on the caudomedial aspect of the ulna. The postoperative caudal radiographic appearance was attributed to chronic ulnohumeral luxation with a poorly defined anconeal process, which led to remodeling of the ulnohumeral joint surface, particularly involving deformation of the trochlear notch of the ulna. The indistinct anconeal process was presumed to be due to osteoarthritic changes, as the fracture line might have involved the process, and chronic non-union over a two-year period likely resulted in its resorption or remodeling. Although imaging demonstrated remodeling and deformation of the trochlear notch consistent with chronic changes, the patient exhibited a normal gait at 140 days postoperatively. The range of motion in the operated limb was mildly reduced but remained within a near-normal range compared to the contralateral side. While objective kinetic assessment such as force plate analysis was not performed, a 5-point lameness scale [19] was used to assess postoperative functional recovery at 2, 6, 12, and 20 weeks by the surgeon who performed the surgery, thereby documenting satisfactory restoration of limb function at the final follow-up (POD 140, scoring 0/5).

## 4. Conclusions

This case report highlights the successful management of a chronic non-union olecranon fracture in a small breed dog through stable internal fixation, and biological enhancement with rhBMP-2-loaded LSS particles and MSCs. The combined approach resulted in satisfactory bone healing and restoration of limb function without the need for arthrodesis. In addition, the radiolucent nature of the LSS scaffold allowed for unobstructed postoperative radiographic evaluation of fracture healing. This strategy may represent a viable option for treating complex, long-standing fractures with compromised healing environments in veterinary patients.

## Data Availability

All data generated or analyzed during this study are included in this published article. Further inquiries can be directed to the corresponding authors.
